# Dissection of the Structural Features of a Fungicidal Antibody-Derived Peptide

**DOI:** 10.3390/ijms19123792

**Published:** 2018-11-28

**Authors:** Thelma A. Pertinhez, Tecla Ciociola, Laura Giovati, Walter Magliani, Silvana Belletti, Luciano Polonelli, Stefania Conti, Alberto Spisni

**Affiliations:** 1 Department of Medicine and Surgery, University of Parma, 43126 Parma, Italy; thelma.pertinhez@unipr.it (T.A.P.); tecla.ciociola@unipr.it (T.C.); laura.giovati@unipr.it (L.G.); walter.magliani@unipr.it (W.M.); silvana.belletti@unipr.it (S.B.); luciano.polonelli@unipr.it (L.P.); alberto.spisni@unipr.it (A.S.); 2 Transfusion Medicine Unit, Azienda USL-IRCCS di Reggio Emilia, 42123 Reggio Emilia, Italy

**Keywords:** antibody-derived peptides, antifungal peptides, cell penetrating peptides, circular dichroism, nuclear magnetic resonance spectroscopy, confocal microscopy, scanning electron microscopy

## Abstract

The synthetic peptide T11F (TCRVDHRGLTF), derived from the constant region of human IgM antibodies, proved to exert a significant activity in vitro against yeast strains, including multidrug resistant isolates. Alanine substitution of positively charged residues led to a decrease in candidacidal activity. A more dramatic reduction in activity resulted from cysteine replacement. Here, we investigated the conformational properties of T11F and its alanine-substituted derivatives by circular dichroism (CD) and nuclear magnetic resonance (NMR) spectroscopy. Peptide interaction with *Candida albicans* cells was studied by confocal and scanning electron microscopy. T11F and most of its derivatives exhibited CD spectra with a negative band around 200 nm and a weaker positive band around 218 nm suggesting, together with NMR coupling constants, the presence of a polyproline II (PPII) helix, a conformational motif involved in a number of biological functions. Analysis of CD spectra revealed a critical role for phenylalanine in preserving the PPII helix. In fact, only the F11A derivative presented a random coil conformation. Interestingly, the loss of secondary structure influenced the rate of killing, which turned out to be significantly reduced. Overall, the obtained results suggest that the PPII conformation contributes in characterising the cell penetrating and fungicidal properties of the investigated peptides.

## 1. Introduction

In recent years, a number of natural and synthetic peptides have been investigated for their antimicrobial activity in order to select candidate molecules able to overcome microbial resistance to currently available drugs. In depth researches aimed to clarify structure–activity relationships and elucidate the mechanisms of action of antimicrobial peptides pave the way for their potential use in clinical practice, alone or in combination with available drugs [1]. A number of studies also indicate that newly designed, modified or conjugated peptides, and peptidomimetics could provide novel therapeutic approaches for the fight against infectious diseases [2,3,4].

In this regard, our group has been involved for several years in the study of antibody-derived peptides endowed with antimicrobial, antiviral, and immunomodulatory activity [5].

In particular, synthetic peptides, whose sequence derived from the constant region of human antibodies, were reported to display fungicidal activities in vitro and/or in vivo, regardless of the isotype of the originating molecule [6]. Among others, the synthetic 11-residues IgM-derived peptide TCRVDHRGLTF (T11F) proved to be fungicidal in vitro, at micromolar concentration, against several yeast strains, including multidrug-resistant isolates. In addition, it did not exhibit any hemolytic, cytotoxic, or genotoxic activity. T11F is characterized by an alternation of charged and hydrophobic residues throughout its primary sequence with a Cys residue close to the N-terminus. These features are known to be relevant for the biological activity of antimicrobial peptides (AMPs) and, in fact, alanine substitution of the positively charged residues caused a decrease in candidacidal activity, while a more dramatic reduction in activity resulted from cysteine replacement [6].

Here we present the results of further studies on T11F and its alanine-substituted derivatives, aimed to correlate their variable ability to interact with and kill *C. albicans* cells with their conformational properties, investigated by circular dichroism (CD) and nuclear magnetic resonance (NMR) spectroscopy. CD spectroscopy highlighted the propensity, for all peptides with the exception of F11A, to acquire a polyproline II (PPII) helix, a conformational motif known to be involved in a number of biological functions. Analysis of the ^3^J_HN-α_ NMR coupling constants enabled the evaluation of the tendency of each residue to be in that conformation. As for the F11A derivative, presenting a random coil conformation, the loss of secondary structure proved to influence the rate of killing, which turned out to be significantly reduced.

Overall, the obtained results suggest that the PPII conformation contributes to characterise the cell penetrating and fungicidal properties of the investigated peptides.

## 2. Results

### 2.1. Peptides Conformational State

**Figure 1:**
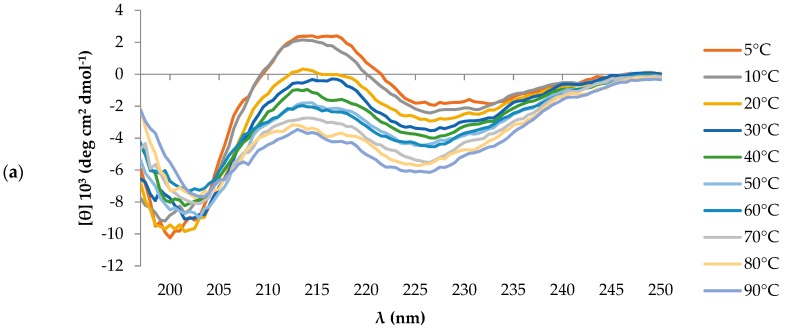

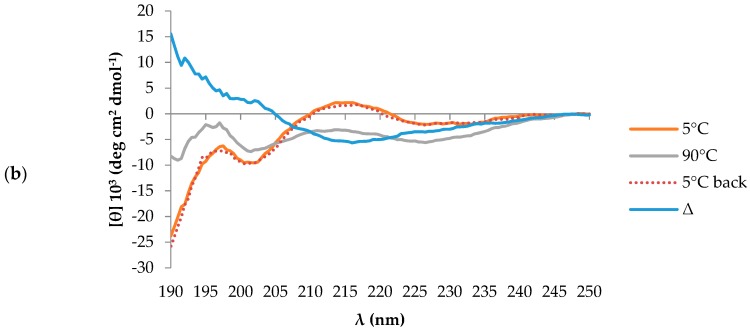
Far-UV CD spectra of 70 μM T11F (TCRVDHRGLTF) obtained immediately after preparation of the starting aqueous solution. (**a**) Spectra acquired starting from 5 °C up to 90 °C; (**b**) T11F thermal denaturation and renaturation. Spectra obtained at 5 °C, after heating at 90 °C, and back to 5 °C. Δ: difference spectrum between 5 °C and 90 °C.

The CD spectrum of T11F, acquired at 5 °C, was characterised by a negative band around 200 nm and by a weaker positive band at ∼218 nm. These spectral features suggested the presence of a PPII helix. Given that the PPII helix is generally more stable at low temperatures, we carried out a set of measurements at increasing temperatures. The decrease of the positive band at 218 nm at higher temperature values (Figure 1a), together with the reversibility of the process observed bringing the temperature back to 5 °C (Figure 1b), supported our hypothesis.

The difference spectrum between the ones obtained at 5 °C and 90 °C, reminiscent of a β-structure (Figure 1b), suggested that heating favours a partial transition of the peptide conformation to a β-structure organisation. Such behaviour has been pointed out by Shi et al. for polypeptides in PPII conformation [7], thus reinforcing the presence of a PPII helix motif in T11F.

**Figure 2:**
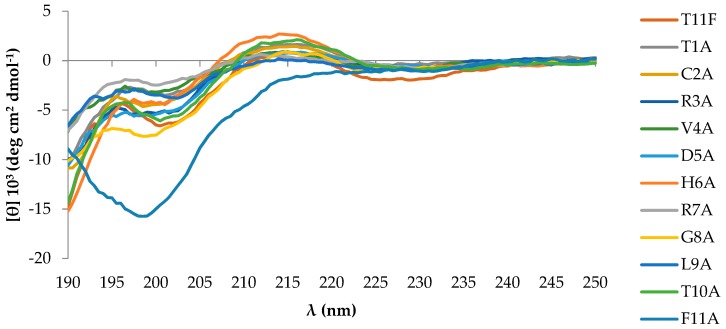
Far-UV CD spectra of 70 μM T11F and its derivatives acquired at 5 °C immediately after preparation of the starting aqueous solution (750 µM).

To evaluate the contribution of each residue in the PPII helix conformation, CD spectra of all alanine-substituted derivatives were acquired at 5 °C and compared with the parental peptide T11F. Only F11A exhibited a random coil conformation (Figure 2), indicating that Phe11 is essential to preserve the PPII conformation. CD spectra were acquired at time 0 and 20 days after solubilisation, during which the aqueous solutions were stored at 4 °C. Spectra acquired after 20 days showed no changes, discarding the onset of any aggregation process or other conformational transitions.

### 2.2. NMR Spectroscopy Characterisation

**Figure 3:**
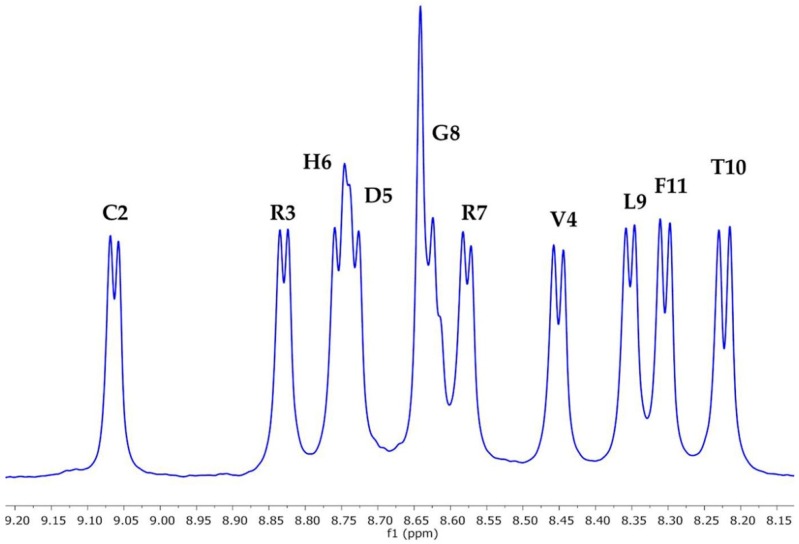
NH region of the ^1^H-NMR spectrum of 3.25 mM T11F in 5% D_2_O/95% H_2_O at pH 5.0.

The NH region of the ^1^H-NMR spectrum of T11F (Figure 3) showed the signals of the 10 backbone NHs as being very well separated, indicating that the peptide has a well-defined secondary structure. Based on the measurable NH-αH coupling constants (^3^J_HN-α_), it was possible to calculate, for each residue, its preference to exist in a PPII conformation (%PPII) [8]; the values are reported in Table 1.

**Figure 4:**
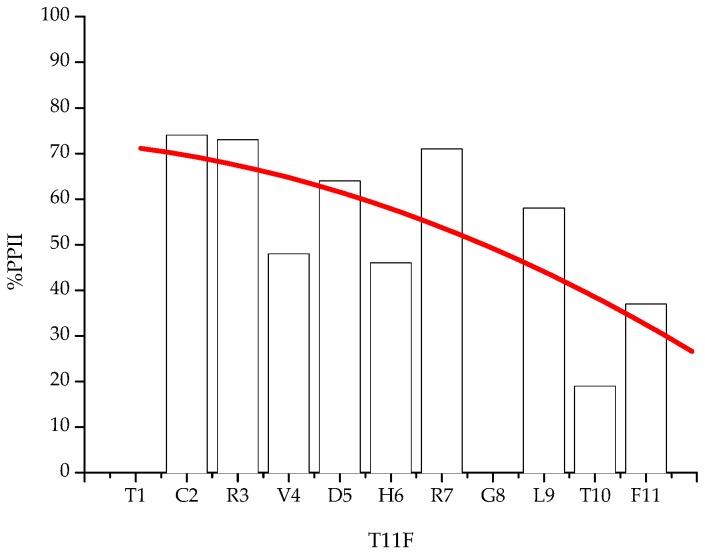
Plot of ^3^J_HN-α_ against T11F primary sequence. ^3^J_HN-α_ for Thr1 and Gly8 are not measurable.

The data indicated that the peptide is characterised by a regular decrease of PPII conformational propensity from the N terminus, where the Cys residue is located, to the C-terminus, where Thr and Phe are located (Figure 4).

This trend is consistent with previous studies pointing to aromatic and bulky, rigid and hydrophobic side chains as being responsible for a destabilisation of this structural motif [8,9].

### 2.3. Time Kinetics of C. albicans Killing

**Figure 5:**
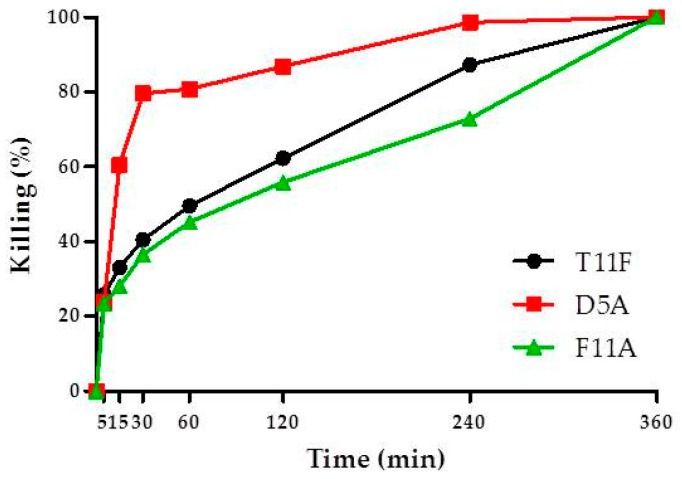
Time kinetics of in vitro activity of selected peptides against *Candida albicans* SC5314 cells. The activity is expressed as percentage killing, reported data represent mean values from three independent experiments (variability ≤10%). Each experiment was performed in triplicate. As determined by two-way ANOVA with Bonferroni post hoc test, significant differences were observed at 15, 30, 60, 120, and 240 min for D5A vs. T11F and F11A (*p* < 0.001 at all times, with the exception of D5A vs. T11F, *p* < 0.05 at 240 min), while a significant difference between T11F and F11A was observed only at 240 min (*p* < 0.01).

To obtain more insights on the molecular mechanisms associated to the biological activity of T11F, we analysed and compared the parent peptide with two of its derivatives. We chose F11A because it was the only one that exhibited a random coil conformation, and D5A because previous data indicated that it is the most active in vitro against the reference *C. albicans* strain, showing a half maximal effective concentration (EC_50_) value of 0.395 × 10^−6^ mol/liter, in comparison to 1.540 × 10^−6^ mol/liter of the parental peptide [6]. A comparison of the rate of killing against *C. albicans* showed that D5A exhibited the more rapid candidacidal effect, achieving 60.5% killing at 15 min and 79.6% killing at 30 min, while the killing was 33.0% and 40.4% for T11F and 28.2% and 36.5% for F11A, at 15 and 30 min, respectively (Figure 5).

### 2.4. Peptide–Yeast Cells Interaction

#### 2.4.1. Confocal Microscopy Studies

**Figure 6:**
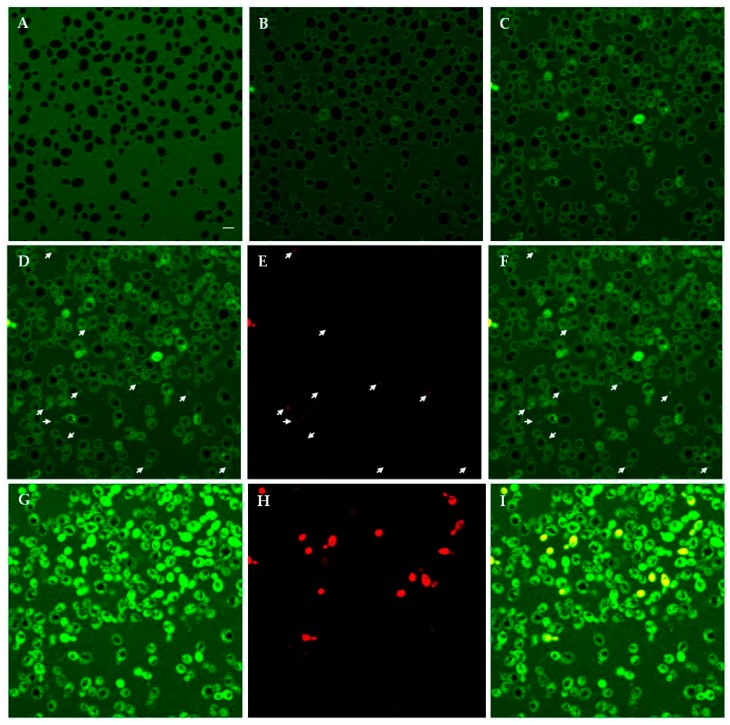
Interaction between *Candida albicans* SC5314 cells and fluorescein isothiocyanate (FITC)-labelled D5A followed in time-lapse confocal microscopy. The same field is shown. Living yeast cells were incubated in the presence of FITC-labelled D5A for 5 min (**A**), 60 min (**B**), 170 min (**C**), 220 min (**D**), and 390 min (**G**). Panels **E** and **H**: PI staining at 220 and 390 min, respectively. Panels **F** and **I**: merge of panels **D** and **E**, and **G** and **H**, respectively. Arrows in panels **D**, **E**, and **F** indicate the leakage of material from yeast cells. Bar = 5 μm.

Confocal microscopy allowed the investigation of the dynamic process of peptide–yeast cells interaction. Fluorescein isothiocyanate (FITC)-labelled T11F, D5A and F11A proved to bind to the yeast cell wall and enter living *C. albicans* SC5314 cells, accumulating inside over time and leading to cell death, as shown by propidium iodide (PI) internalisation. As an example, time-lapse observations of FITC-labelled D5A treated preparation are shown in Figure 6. After 5 min of treatment the peptide localised on the yeast cell wall (panel A). Over time, the peptide entered the yeast cells with a progressive increase of intracellular localisation (panels B, C, D, G). The leakage of cellular material, evidenced by PI binding, was observed (panels D, FITC; E, PI; F, merge of D and E) for D5A and also for T11F (see below). Interestingly, the leakage of cellular material was not observed for F11A. After 390 min a number of dead cells were visible, as demonstrated by the merge of green (FITC) and red (PI) signals (panels G, FITC; H, PI; I, merge of G and H).

**Figure 7:**
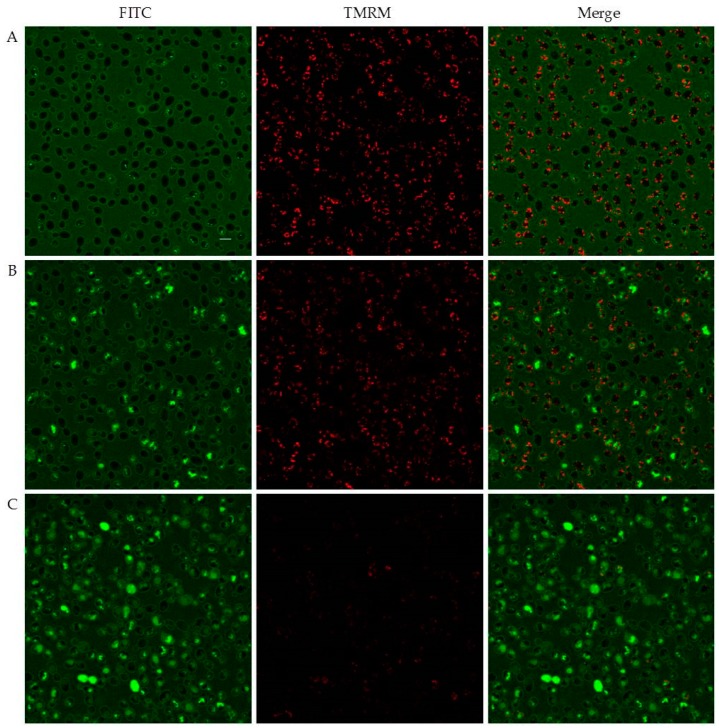
Confocal images of living *Candida albicans* SC5314 cells pre-treated for 1 h with tetramethylrhodamine methyl ester perchlorate (TMRM) after addition of FITC-labelled D5A for 5 min (**A**), 60 min (**B**), and 165 min (**C**). Bar = 5 µm.

In selected experiments, tetramethylrhodamine methyl ester perchlorate (TMRM) was used as mitochondrial probe. The dye was rapidly accumulated into mitochondria of living cells, but its signal significantly decreased after treatment with peptides. As highlighted by merge images, intracellular peptide did not co-localise with TMRM, i.e. did not enter mitochondria. Time-lapse images of TMRM pre-treated yeast cells following addition of FITC-labelled D5A are shown in Figure 7.

#### 2.4.2. Scanning Electron Microscopy (SEM) Studies

**Figure 8:**
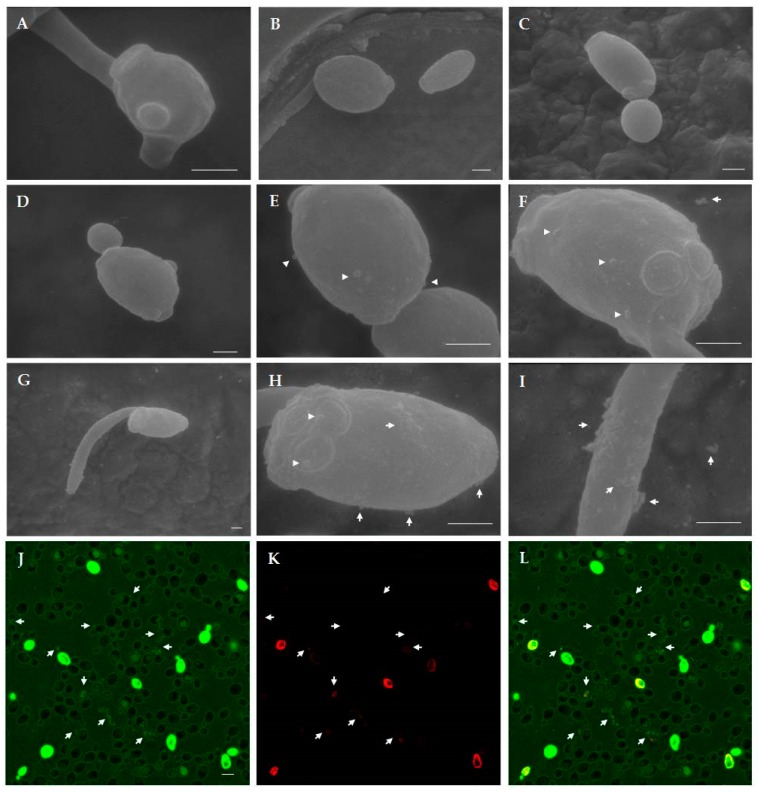
Scanning electron microscopy (SEM) and confocal microscopy images of *Candida albicans* cells treated with T11F. At SEM observation, structural and morphological alterations were evident after treatment with the peptide for 1 h (panels **D**–**I**) in comparison to untreated (control) cells (panels **A**, **B**, **C**). In panels **E**, **F**, **H**, and **I**, arrowheads indicate blebs and arrows indicate the leakage of material from yeast cells. Images in panels **H** and **I** show details of cell in panel **G**. (**A**–**I**: Bar = 1 µm). Confocal microscopy images after T11F treatment showed the leakage of material from yeast cells, indicated by arrows (panel **J**, FITC; panel **K**, PI; panel **L**, merge). (**J**–**L**: Bar = 5 µm).

SEM observation revealed structural and morphological alterations in *C. albicans* cells treated with T11F in comparison to control cells (Figure 8). In particular, while untreated control cells (panels A–C) presented a round and compact structure, after peptide treatment a loss of cell turgidity was evident and the cell surface appeared irregular (panels D–I). The presence of blebs and a leakage of cellular material was also visible (panels E, F, H, and I), matching the observations by confocal microscopy (panels J–L).

### 2.5. Evaluation of Apoptosis in C. albicans Cells after Treatment with Selected Peptides

**Figure 9:**
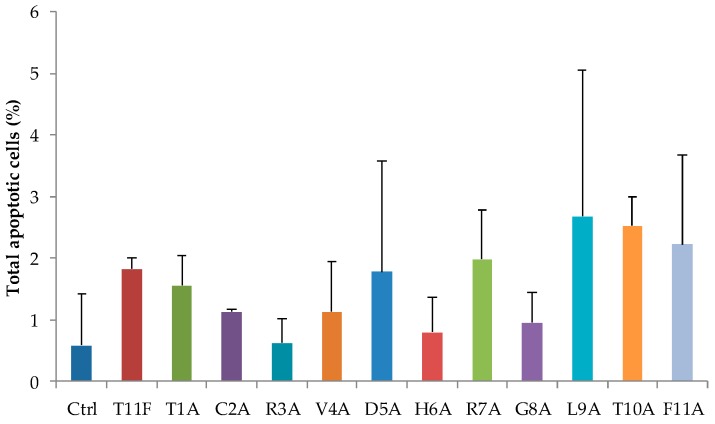
Lacking of apoptotic effects of T11F and its derivatives in *Candida albicans* SC5314 cells. Phosphatidylserine externalisation was analysed by flow cytometry, after 30 min of treatment with peptides at their EC_50_ value. Data represent the mean ± standard deviation of at least three independent experiments.

Phosphatidylserine externalisation and reactivity with annexin V was determined by flow cytometry in order to assess if the peptides, at their EC_50_ values, could induce apoptosis in *C. albicans* SC5314 whole cells. Under the experimental conditions adopted, none of the peptides proved to induce apoptosis. In fact, no statistically significant difference was found between the percentage of apoptotic cells in the absence (control) or in the presence of peptides (Figure 9).

## 3. Discussion

T11F is a peptide that exhibits antimicrobial activity and that, in solution, exists in a dimeric form stabilised by the presence of a Cys residue at the N-terminus and by the regular alternation of charged and uncharged residues [6]. Alanine scanning is a standard and systematic approach to identify the contribution of amino acid residues to the peptide’s function and conformation [10,11]. Previous studies showed that alanine substitution of the positively charged residues caused a decrease in candidacidal activity, while a more dramatic reduction in activity resulted from cysteine replacement [6]. These results prompted us to investigate a possible correlation between the variable biological activity of T11F and its alanine-substituted derivatives and their conformational features.

CD spectroscopy revealed the ability of T11F to acquire a left-handed 3_1_-helix conformation (PPII helix) in aqueous solution (Figure 1). In fact, the CD spectrum of a pure polyproline peptide in PPII helix conformation shows distinctive features consisting of a weak positive band at 229 nm associated with a more intense negative band around 196–198 nm [12]. However, when other residues are present in the primary sequence the positive band shifts towards shorter wavelengths [13] in the range 210–230 nm [14] as in the case of T11F.

Because it is well accepted that PPII conformation is involved in a number of biological functions of peptides and proteins [15,16], we decided to investigate the possible role of that conformational motif in the biological activity of the peptide.

The NMR results showed that all residues exist in a conformation that, to a variable extent, contributes to the stabilisation of a PPII conformation. Interestingly, residues with higher propensity for a PPII conformation are found in the N-terminus region, where also the Cys residue is located, while residues less keen to exist in PPII conformation are located in the C-terminus portion (Figure 4).

Overall, these data suggest that T11F in solution forms a dimeric structure with a PPII fold.

**Figure 10:**
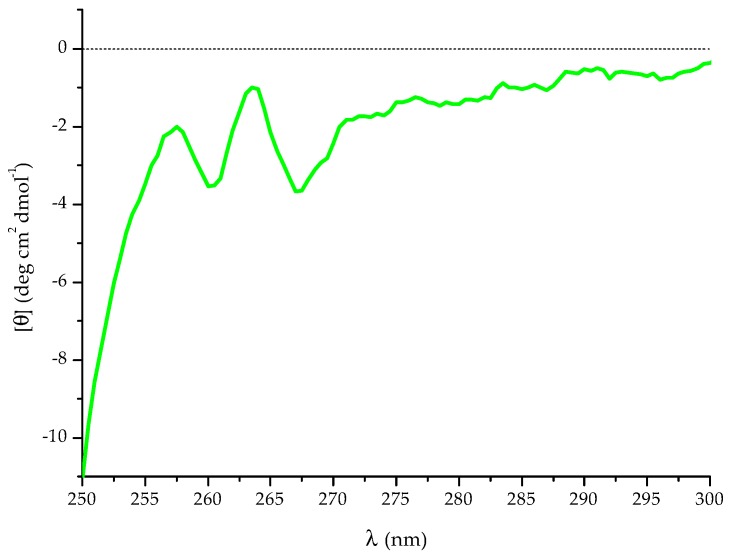
Near-UV CD spectrum of 3.25 mM T11F at 5 °C.

The CD spectra of T11F alanine-substituted derivatives are quite similar (Figure 2), with the only exception being the F11A derivative which exhibits a complete loss of ordered conformational organisation. An explanation of this fact may be found by observing that the T11F near-UV CD spectrum (Figure 10) presents two bands in the range 260–265 nm, highlighting the interaction between the Phe aromatic rings.

We can envisage that the T11F dimeric structure is stabilised on one side by the formation of the Cys disulfide bridge and on the other by the π–π interaction between the Phe11 side chain of each monomer. Substitution of Phe11 with Ala removes the interaction favouring backbone–solvent interaction thus destabilising the peptide secondary structure. The fact that C2A maintains a PPII conformation is justified by recognizing that, differently from the C-terminus region, in the N-terminus there are residues with a clear tendency to stabilise the PPII conformation. Therefore, even in the absence of Cys2 the ordered secondary structure of the dimer is maintained.

Apparently, these structural features do not seem to be involved in the biological activity of the peptides as T11F and the F11A derivative are both able to kill *C. albicans* [6]. However, if we analyse the rate of killing (Figure 5), we may observe a difference for the three selected peptides, with D5A being faster and F11A exhibiting the lower rate. The higher rate of killing found for D5A is explained by its high positive charge (+3), that boosts the initial binding to the cellular membrane, the first step of the interaction between peptide and yeast cells (Figure 6). Subsequently, (as shown in Figure 6, Figure 7 and Figure 8) peptide penetration and finally leakage of cellular material are observed. Interestingly, this last phenomenon, i.e., the leakage of cellular material, is not observed after treatment with F11A that, however, is able to kill *Candida* cells. If we combine this fact with the slower rate of killing, it is feasible to hypothesise that the lack of PPII structure renders the interaction of the peptide less efficient in terms of binding, penetration and finally of inducing a membrane disruption sufficient for a leakage of cellular material. Results obtained with other peptides [17,18] highlight the critical role of Phe residues, in AMPs, in terms of facilitating their membrane binding and permeabilisation action. Interestingly, in those cases, the authors focused on the hydrophobic and aromatic features of the Phe side chain as key factors.

In the case of T11F we may expect that Phe acts by combining, in a synergic fashion, all its features.

Overall, we can conclude that the ability to acquire a PPII conformation is one of the factors that contribute to define the cell penetrating and fungicidal properties of the investigated peptides.

More at large, these data support a stimulating view: accepting the notion that the ability of these peptides to bind and penetrate the cellular membrane is only part of their mechanism of action, and recognizing that indeed they may activate other processes when inside the cell, as observed with other peptides [19,20,21], we can envisage new criteria to design membrane disrupting peptides with pharmacological applications both in medical and dental practice.

## 4. Materials and Methods

### 4.1. Peptide Synthesis

T11F peptide (TCRVDHRGLTF) and its alanine-substituted derivatives were synthesised at CRIBI-Peptide Facility (University of Padua, Italy) by the fluorenylmethyloxycarbonyl (Fmoc) solid-phase synthesis chemistry, as previously described [22]. Peptides were purified by preparative reverse-phase high-performance liquid chromatography (HPLC) and their molecular mass was determined by mass spectroscopy. Peptide purity was in the range of 80%–90% as measured by analytical reverse phase HPLC.

### 4.2. CD Spectroscopy

CD studies were performed by using a Jasco 715 spectropolarimeter (JASCO International Co. Ltd., Tokyo, Japan), coupled to a Peltier PTC-348WI system for temperature control, as previously described [22]. Far-UV spectra were recorded at different temperatures (5–90 °C) in the range 250–190 nm, using a 1 mm path length quartz cuvette. Peptides (at a final concentration of 70 µM) were analysed immediately or at different time points after preparation of the starting aqueous solution (750 µM). Thermal denaturation of the T11F peptide was monitored from 5–90 °C with a temperature increase of 1 °C/min. Near-UV CD of 3.25 mM T11F was acquired from 250 to 300 nm, using a 0.5 cm cell at 5 °C. The spectra were water baseline corrected and the spectral intensity was converted to molar mean residue ellipticity [θ] (deg cm^2^ dmol^−1^).

### 4.3. NMR Spectroscopy

T11F was dissolved in 600 μL of a 5% D_2_O/95% H_2_O mixture to yield a final concentration of 3.25 mM at pH 5.0. The ^1^H NMR spectra were recorded using a Varian INOVA 600AS spectrometer at 5 °C. Two-dimensional proton homonuclear experiments TOCSY (70 ms mixing time), NOESY (150 and 200 ms mixing times) and ROESY (100, 300 ms mixing time) were performed using standard pulse sequences [23]. ^1^H and ^15^N chemical shift assignments are reported in Table S1 (supplementary material). The ^3^J_NH-α_ coupling constants were measured from 1D spectra (128 k data points, no line broadening) using the software MestReNova 11. Data were processed using NMRPipe/NMRView algorithms [24,25]. The PPII population (%PPII) for each residue of T11F was calculated with the Equation (1):

%PPII × J_PPII_ + (1 − %PPII) × J_β_ = J_T11F_ (1)

where J_PPII_ and J_β_ are the reference ^3^J_NH-α_ values for PPII and β conformations of each amino acid, respectively [8].

### 4.4. Time Kinetics of Peptide-Mediated Killing of C. albicans

Time kinetics of in vitro activity of T11F and selected derivatives against *C. albicans* SC5314 was evaluated, at different times (5, 15, 30, 60, 120, 240, and 360 min) by colony forming unit (CFU) assays, as previously described [26]. The selected peptides were used at their minimal fungicidal concentration, previously assessed by CFU assays, i.e., 5 μg/mL for T11F, 2 μg/mL for D5A and 10 μg/mL for F11A. Each CFU assay was performed in triplicate. The activity was expressed as percent killing, calculated as: 100−(average number of CFU in the peptide-treated group/average number of CFU in the control group) ×100. Reported data represent the average from three independent experiments.

### 4.5. Confocal Microscopy Studies

Confocal microscopy studies were carried out by an LSM 510 Meta scan head integrated with the Axiovert 200 M inverted microscope (Carl Zeiss, Jena, Germany) as previously described [22]. Briefly, 20 μL from a suspension of 2 × 10^7^ *C. albicans* SC5314 cells/mL were seeded on coverslips mounted in a special flow chamber. A selected field was kept and observed along the time lapse experiment. After 30 min, the peptide labelled with FITC as previously described [22] was added (final concentration 150 μM). Propidium iodide (PI), a non-vital nuclear stain commonly used for identifying dead cells, was added (1.5 μM) after 30 min. Images were taken after FITC-labelled peptide addition up to 390 min. In selected experiments, 2 × 10^7^ cells/ml prepared as above described were loaded with 200 nM TMRM (Invitrogen, Molecular probes, Eugene, OR, USA) for 1 h at room temperature in the dark. Then, 20 μL of suspension were seeded on coverslips and after 30 min the labelled-peptide was added (final concentration 150 μM) and TMRM, FITC, and PI fluorescence observed along the time. PI and TMRM were excited with 543 nm He-Ne laser line and FITC with 488 nm argon laser line. Acquisition was carried out in a multitrack mode (namely through consecutive and independent optical pathways).

### 4.6. SEM Studies

For SEM studies, *C. albicans* SC5314 cell suspensions prepared as previously described [27], were incubated for 1 h in the absence (control) or presence of T11F (190 µM). Samples of 5 µL of the suspensions were placed on 25 mm^2^ glass slides, fixed with glutaraldehyde in sodium cacodylate, then prepared as previously described [22] for microscopic examination by a Philips 501 scanning electron microscope (15 kV).

### 4.7. Evaluation of Apoptosis Induction Profiles in C. albicans after Treatment with Selected Peptides

Peptide-induced effects in *C. albicans* SC5314 cells were evaluated by the Muse Cell Analyzer (Merck Millipore, Darmstadt, Germany) using the Muse Annexin V & Dead Cell Assay kit, based on the detection of phosphatidylserine on the surface of apoptotic cells, as previously described [22]. Yeast cells suspended in water (5 × 10^5^ cells/mL) were maintained for 30 min in the presence or absence (control) of selected peptides at their EC_50_. For the evaluation of the apoptotic profile, after addition of the proper reagents, data were acquired according to the manufacturer’s instructions.

## Figures and Tables

**Table 1:** ^3^J_HN-α_ coupling constants (Hz) for T11F, reference ^3^J_HN-α_ values for PPII and β-sheet, and the PPII calculated population (%PPII) for each residue.

Residue	^3^J_HN-α_ T11F	^3^J_HN-α_ * (PPII)	^3^J_HN-α_ * (β-sheet)	%PPII
**Thr1**	−	6.06	9.62	−
**Cys2**	6.92	5.93	9.70	74
**Arg3**	6.87	5.89	9.51	73
**Val4**	8.00	6.09	9.82	48
**Asp5**	7.16	5.72	9.69	64
**His6**	7.67	5.65	9.41	46
**Arg7**	6.92	5.89	9.51	71
**Gly8**	nd	−	−	nd
**Leu9**	7.13	5.60	9.24	58
**Thr10**	8.93	6.06	9.62	19
**Phe11**	8.19	5.34	9.86	37

* Reference values for ^3^J_HN-α_ for PPII and β-sheet.

## Supplementary Materials

Supplementary materials can be found at http://www.mdpi.com/1422-0067/19/12/3792/s1.

## Abbreviations

CD	circular dichroism
CFU	colony forming unit
FITC	fluorescein isothiocyanate
HPLC	high-performance liquid chromatography
NMR	nuclear magnetic resonance
PI	propidium iodide
SEM	scanning electron microscopy
TMRM	tetramethylrhodamine methyl ester
