# Supplementary material for: Dissection of the Structural Features of a Fungicidal Antibody-Derived Peptide
Source: Int J Mol Sci. 2018 Nov 28;19(12):3792. doi: 10.3390/ijms19123792 (PMC6321458; doi:10.3390/ijms19123792)
Supplement: Supplementary file 1 [file ijms-19-03792-s001.pdf]

Supplementary Material

Table S1. ^{1}H-NMR and ^{15}N NMR chemical shifts (ppm) for T11F in 95% H_2O/5% D_2O, pH 5.0 at 5 °C.

Residue	^{15}N	NH	αH	βH	γH	Others
**Thr1**	-	-	4.17	3.97	1.37	-
**Cys2**	123.6	9.07	4.62	2.98	-	-
**Arg3**	125.0	8.83	4.41	1.87,1.81	1.69,1.63	δH 3.32; NH 7.32
**Val4**	122.4	8.45	4.12	2.02	0.96,0.87	-
**Asp5**	123.7	8.74	4.70	2.88,2.79	-	-
**His6**	119.9	8.75	4.73	3.21,3.34	-	2H 8.65; 4H 7.33
**Arg7**	122.3	8.58	4.35	1.90,1.84	1.67	δH 3.22; NH 7.32
**Gly8**	110.1	8.63	4.01	-	-	-
**Leu9**	121.3	8.35	4.45	1.69,1.66	1.55	δH 0.96,0.92
**Thr10**	114.3	8.22	4.37	4.20	1.21	-
**Phe11**	124.0	8.31	4.66	3.23,3.07	-	2,6H 7.28, 3,5H 7.34
